# RNA G-quadruplex structures control ribosomal protein production

**DOI:** 10.1038/s41598-021-01847-6

**Published:** 2021-11-23

**Authors:** Dhaval Varshney, Sergio Martinez Cuesta, Barbara Herdy, Ummi Binti Abdullah, David Tannahill, Shankar Balasubramanian

**Affiliations:** 1 Cancer Research UK Cambridge Institute, Li Ka Shing Centre, Robinson Way, Cambridge, CB2 0RE UK; 2 Yusuf Hamied Department of Chemistry, University of Cambridge, Cambridge, CB2 1EW UK; 3 School of Clinical Medicine, University of Cambridge, Cambridge, CB2 0SP UK; 4 Present Address: Data Sciences and Quantitative Biology, Discovery Sciences, AstraZeneca, Cambridge, UK; 5 Present Address: Weatherall Institute of Molecular Medicine, University of Oxford, Oxford, UK

**Keywords:** Translation, Nucleic acids, Chemical biology

## Abstract

Four-stranded G-quadruplex (G4) structures form from guanine-rich tracts, but the extent of their formation in cellular RNA and details of their role in RNA biology remain poorly defined. Herein, we first delineate the presence of endogenous RNA G4s in the human cytoplasmic transcriptome via the binding sites of G4-interacting proteins, DDX3X (previously published), DHX36 and GRSF1. We demonstrate that a sub-population of these RNA G4s are reliably detected as folded structures in cross-linked cellular lysates using the G4 structure-specific antibody BG4. The 5′ UTRs of protein coding mRNAs show significant enrichment in folded RNA G4s, particularly those for ribosomal proteins. Mutational disruption of G4s in ribosomal protein UTRs alleviates translation in vitro, whereas in cells, depletion of G4-resolving helicases or treatment with G4-stabilising small molecules inhibit the translation of ribosomal protein mRNAs. Our findings point to a common mode for translational co-regulation mediated by G4 structures. The results reveal a potential avenue for therapeutic intervention in diseases with dysregulated translation, such as cancer.

## Introduction

RNA G-quadruplexes (G4s) are thought to influence diverse aspects of biology that include transcription, splicing, sub-cellular localisation, translation and decay^1^. Evidence from chemical biology and genetic experiments has accumulated in support of a role for RNA G4s in translational regulation of disparate mRNAs such as NRAS^2^, BCL2^3^ and ADAM10^4^ in vitro and in cells. Potential formation of RNA G4s in thousands of transcripts has been demonstrated in vitro using transcriptome-wide approaches, including reverse transcriptase (RT) stalling based rG4-seq^5^, and SHALiPE^6^ which detects differences in acylation kinetics of 2′-hydroxyl groups. Imaging experiments using a G4 structure-specific antibody, as well as application of small molecule stabilising ligands to cells, have provided significant evidence for the *bona fide* cellular existence of RNA G4s^7^. Experiments to chemically map RNA structure formation in cells by DMS and SHAPE have suggested that RNA G4s may be largely unfolded due to the activity of cellular proteins^8^. In contrast, a recent in-situ chemical mapping study using azido-kethoxal in combination with RT-stalling provided evidence for the formation of RNA G4s in cells^9^. The ability of such methods to detect G4 structures is somewhat limited as they can alter the equilibrium by chemically trapping dynamic structures in an unfolded state and therefore the reaction conditions need to be carefully optimised to sample dynamic or lowly populated structures (see^10^ for more extensive discussion).

The identification of numerous proteins that unwind G4s in RNA, suggests that cells have mechanisms to actively suppress these structures^1,11^. For example, the abundant DHX36 RNA helicase has sub-nanomolar affinity for RNA G4s^12^. Mechanistic studies show that DHX36 binds a folded G4 structure to unwind it in an ATP-independent manner, but can remain bound to facilitate G4 refolding in an ATP-dependent step-wise process^13,14^. Multiple rounds of folding and unfolding can occur prior to dissociation of the enzyme. This indicates that, RNA G4s, much like DNA G4s^15^, undergo dynamic interconversion by associated proteins. Many roles for G4s have been inferred in RNA biology, but critical information regarding their abundance, dynamics and locations within the cellular transcriptome is lacking. Herein, we present evidence for frequent G4 formation in the cellular transcriptome through experiments that exploit endogenous RNA binding proteins and also an engineered G4 structure-specific antibody. Our data reveal many G4s that have hitherto been unrecognised in the endogenous transcriptome and highlight a striking enrichment of G4s in a subset of mRNAs essential for ribosomal assembly. Through chemical biology interventions, we demonstrate a direct role for RNA G4s in the regulation of cellular ribosomal protein production and discuss their possible role in regulating global translation.

## Results

### DDX3X, DHX36 and GRSF1 bind RNA G4 motifs

To generate a comprehensive map of the RNA G4 structures in cells, we first mapped G4s via their recognition by multiple proteins that have been documented to specifically interact with RNA G4 structures^11^. We previously used RNA crosslinking and affinity enrichment (termed iCLAE) to demonstrate that DDX3X binds RNA G4s in cells via its GAR domain^11^. To generate a more comprehensive cellular map of RNA G4s, we have generated additional data for DHX36 and GRSF1 using the same cell line (HEK293 Flp-In T-REx) and iCLAE methodology^11^. Briefly, expression of Streptactin/haemaglutinin (ST/HA)-tagged DHX36/GRSF1 was induced to reflect endogenous levels of protein expression (Fig. S1a). In agreement with previous reports^16,17^, immunofluorescence confirmed the cytoplasmic localisation of tagged proteins (Fig. S1b). Cytoplasmic RNA-protein complexes were isolated from nitrocellulose membranes (Fig. S1c) and cDNA synthesis performed in lithium buffers to prevent RT stalling at G-rich regions^5^.

The RNA helicase DDX3X selectively binds the NRAS RNA G4 in vitro (Kd = 18 nM for NRAS RNA G4) with minimal binding to the mutated G4 (Fig. S2a). Our existing DDX3X dataset (GSE106476) was realigned to human genome (*hg38*) alongside the DHX36 and GRSF1 iCLAE data for consistency. We have previously described DDX3X iCLAE data in detail^11^, but briefly, 4557 consensus peaks from three independent replicates of DDX3X iCLAE (Pearson’s correlation > 0.74) showed significant enrichment in 5′ UTRs (fold change > 45-fold; FDR < 1 × 10^−4^) of mRNA (Fig. S2b). Three tetrad G4s represent 16% of DDX3X iCLAE peaks, which is 4.3-fold higher than what would be expected upon sampling from the transcriptome at random (FDR < 1 × 10^−4^). Two-tetrad G4s comprise the majority (60%) of peaks, which is 2.9-fold greater than random (FDR < 1 × 10^−4^). Since two-tetrad motifs are more prevalent across the transcriptome when compared to three-tetrads, this results in a greater chance of being sampled at random and therefore a lower fold change.

**Figure 1:**
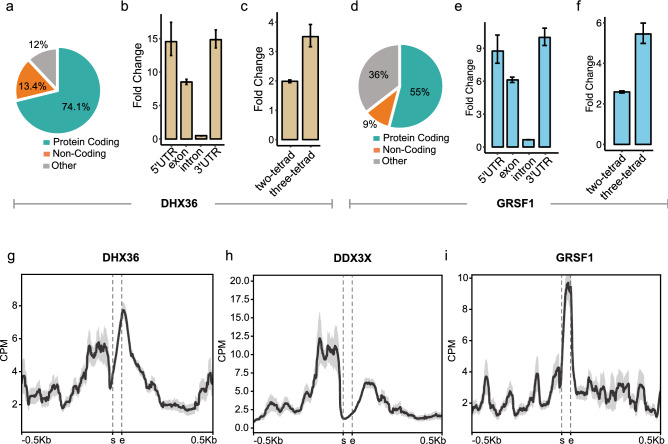
RNA binding proteins interact with G4s in mRNA. (**a,d**) Incidence of iCLAE peaks within protein-coding, non-coding or unannotated regions (other) of the genome. (**b,e**) Enrichment of iCLAE peaks at annotated RNA features. (**c,f**) Enrichment of iCLAE peaks at predicted G4 motifs. Enrichment calculated following random shuffling of peaks in the transcriptome (FDR = 1 × 10^−4^). Error bars represent 95% confidence interval. (**g–i**) Distribution of DHX36, DDX3X and GRSF1 iCLAE reads relative to G4 motifs. Graphs denote pileups of iCLAE reads from multiple sites in CPM. DDX3X iCLAE data has been previously published^11^.

In vitro, recombinant DHX36 demonstrates sub-nanomolar affinity for RNA G4s (Kd = 0.9 nM against NRAS RNA G4 (Fig. S2c)). Three independent iCLAE replicates showed good signal correlation (Pearson’s correlation > 0.98; Fig. S3a), whereas little correlation (Pearson’s correlation < 0.23; Fig. S3a) was observed between DHX36 binding and RNA-seq signal, ruling out non-specific interactions. Overlapping peaks from iCLAE replicates identified 10,891 consensus-binding sites for DHX36 (Fig. S3a, Data S1) and shows significant enrichment (> 25-fold, FDR < 1 × 10^−4^) over previously published PAR-CLIP peaks for DHX36 (Fig. S4a)^16^. The majority (74.1%) of these peaks were found in 3226 protein coding mRNAs (Fig. 1a) that include previously identified target RNA such as NKX2-5^18^ and PITX1^19^ (Fig. S5a). Peaks were particularly enriched within the 5′ and 3′ UTRs (p-value < 0.05; Fig. 1b), which agrees with enrichment analysis of previously published DHX36 PAR-CLIP sites (Fig. S4b)^16^. Motif analysis of DHX36 binding sites in 5′ UTRs primarily show enrichment for G4s (e-value < 1.3 × 10^−45^), whereas in 3′ UTRs an AU-rich motif was more prevalent (e-value < 8.5 × 10^−61^; 58% of peaks in 3′ UTRs, Fig. S5b). This association with AU-rich RNA is consistent with DHX36 also being identified as an AU-rich binding protein^20^ and was also highlighted in previous DHX36 PAR-CLIP data^16^. Confirming that DHX36 targets RNA G4s in cells, the majority of peaks (> 65%) were found to overlap with G4 motifs (Fig. S5c), of which many (> 24%) have been previously confirmed to fold in vitro by rG4-seq^5^. Moreover, these binding sites demonstrated significant enrichment for G4 motifs (Fig. 1c; FDR < 1 × 10^−4^; three-tetrad G4s > 3.5-fold; two-tetrad G4s > 2-fold) and a greater enrichment of rG4-seq verified G4s (FDR < 1 × 10^−4^; > 18-fold) when compared to random.

We previously identified GRSF1 as a *bona fide* interactor of the RNA G4 from NRAS 5′ UTR^11^. GRSF1 has also been reported to specifically interact with RNA G4s in vitro, where binding is suggested to promote G4 unfolding^17^. A role for GRSF1 as a G4 surveillance factor that mediates the decay of mitochondrial non-coding RNAs has been suggested^17^. Four independent iCLAE replicates showed good signal correlation (Pearson’s correlation > 0.99; Fig. S3b), with little correlation (Pearson’s correlation < 0.07; Fig. S3b) between GRSF1 binding and RNA-seq signal, ruling out non-specific interactions. We identified 16,572 consensus GRSF1 binding sites from four independent biological replicates (Pearson’s correlation > 0.94; Fig. S3b, Data S1), which show good overlap with GRSF1 binding sites identified previously by eCLIP (ENCFF929AWR). Known mitochondrial target RNAs of GRSF1, such as COX genes and RMRP, show clear binding sites that overlap with G4 motifs (Fig. S5d)^21^. However, most iCLAE peaks (99%) map to nuclear chromosomes with 55% located in 4228 protein-coding mRNAs, strongly suggesting a non-mitochondrial role for GRSF1 (Fig. 1d). GRSF1 binding also shows a greater enrichment over the UTRs, albeit to a lesser extent than seen for DHX36 (Fig. 1e, p-value < 0.05). Meme motif analysis reveals GRSF1 binds G4 motifs both in 5′ (e-value = 7.3 × 10^−31^) and 3′ UTRs (e-value = 5.2 × 10^−72^; Fig. S5e) and is in contrast with DHX36 which binds AU-rich RNA in 3′ UTRs. Supporting G4 binding in cells, the majority of GRSF1 binding sites (> 77%) overlapped with predicted G4 motifs (Fig. S5f), of which many (> 17%) have been previously confirmed to fold in vitro by rG4-seq^5^. GRSF1 binding sites showed significant enrichment (FDR < 1 × 10^−4^) for G4 motifs (Fig. 1f; three-tetrad G4s > 5-fold and two-tetrad G4s > 2.5-fold) and a greater enrichment of rG4-seq verified G4s (FDR < 1 × 10^−4^; > 18-fold) than random.

Both DHX36 and GRSF1 interact with thousands of two-tetrad G4s (Fig S5c,f), similar to observations for DDX3X (Fig. S2b)^11^. Therefore, our iCLAE data confirms that two-tetrad G4s form in the human transcriptome, in line with other in vitro^22,23^, and in vivo reports^24–27^. DDX3X, DHX36 and GRSF1 bind primarily to distinct target sites (Fig. S6a), with 909 sites overlapping between all three proteins. Sites that are recognised by more than one protein show a greater proportion of predicted G4s compared to sites unique to individual proteins (Fig. S6b), and therefore may represent more persistent G4 structures.

### Functional interaction of proteins with RNA G4s

Data from phase III of the Encode project indicates that the cellular RNA target sites for many RNA binding proteins are defined by their in vitro binding specificities^28^. Likewise, all three proteins studied here, DDX3X^11^, DHX36 and GRSF1, demonstrate binding to G4s in vitro and show enrichment of G4 motifs within their cellular targets. The exact positioning of iCLAE reads relative to the target sites can reveal details of how a protein interacts with its targets^29^. We observed that DHX36 iCLAE reads displayed a 3′ skew (Fig. 1g), which is in accord with biophysical measurements showing DHX36 association with the 3′ single-stranded tail of a G4^12,14^. In contrast, DDX3X iCLAE reads displayed a significant 5′ skew (Fig. 1h), which agrees with in vitro observations of DDX3X preference for 5′ single stranded overhangs^30^. GRSF1 reads were centred at the G4 motif (Fig. 1i) and is consistent with biophysical data showing GRSF1 binding to G-rich sequences^17^. Thus, in each case the cellular binding sites of proteins with respect to G4 motifs show significant differences from each other (p-value < 2.2 < 10^−16^) and faithfully reflect the corresponding biophysical observations.

### Detection of endogenous RNA G4 by a structure specific antibody

**Figure 2:**
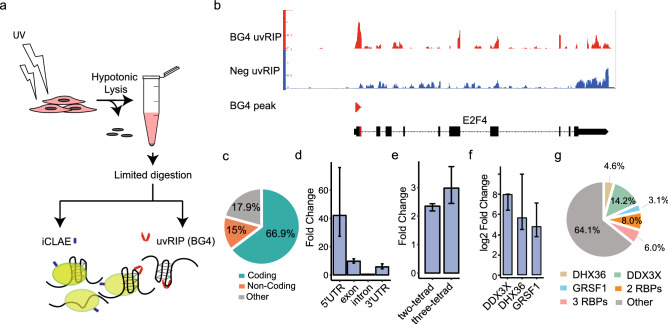
Identification of cellular G4s using BG4 uvRIP. (**a**) Experimental strategy. (**b**) Example snapshot of BG4 uvRIP peak in the 5′ UTR of E2F4 gene. A negative control is provided by uvRIP using a non-G4 specific A9 antibody. (**c**) Overlap of BG4 uvRIP peaks with protein-coding, non-coding or unannotated regions of the genome. (**d**) Enrichment of BG4 uvRIP peaks within annotated RNA features. (**e**) Enrichment of G4 motifs within BG4 uvRIP peaks. (**f**) Enrichment of BG4 uvRIP peaks within iCLAE sites for DDX3X, DHX36 and GRSF1. For (**d–f**) enrichment calculated following random shuffling of peaks in the transcriptome. (**g**) Percentage overlap of BG4 uvRIP peaks with iCLAE datasets for DDX3X, DHX36 and GRSF1.

To provide cross-validation for the formation of RNA G4s in mRNAs by an orthogonal approach, we performed affinity enrichment experiments with the well-characterised G4-structure specific single chain antibody BG4^7^. BG4 shows high affinity for G4 structures (Kd ~ low nM) with little cross-reactivity to linear or non-G4 nucleic acid structures^31^. We first UV-crosslinked cells to trap RNA-protein complexes in their endogenous states, followed by limited RNA fragmentation using RNaseA and then finally BG4 affinity capture of folded G4 structures (Fig. 2a). This cross-linked RNA immunoprecipitation (uvRIP) method enriches for RNA fragments displaying accessible G4s, which are not unfolded or masked by proteins. In parallel, we also performed uvRIP using a negative control antibody (A9), which has a closely related single-chain scaffold to BG4, but does not bind G4s. Overall, we identified 1428 consensus sites across three independent replicates that were significantly enriched by BG4 uvRIP compared to size matched inputs (FDR < 0.05), and not enriched in the negative control uvRIP. These sites, hereafter referred to as BG4 peaks, showed a median size of 174 nt, with 90% of the peaks being smaller than 400 nt (example snapshots shown in Fig. 2b, Supplementary Fig. S7). BG4 peaks were highly enriched in 5′ UTRs (42-fold when compared to random shuffled, FDR < 0.001), and to a lesser extent in the exons (tenfold, FDR < 0.001) and 3′ UTRs (sevenfold, FDR < 0.001) of mRNA (Fig. 2c,d). Our detection of folded G4s in UTRs is in keeping with computational predictions that highlight the prevalence of G4s in UTRs^32^, and also with experimental data from in vitro polymerase^33^ and reverse transcriptase-stalling assays^5^. Most (65%) RNA G4s previously identified in purified, protein-free transcripts by reverse transcriptase-stalling (rG4-seq) were in 3′ UTRs with enrichment near polyadenylation signals^5^, whereas in a cellular context BG4 uvRIP experiments revealed that only 7.5% of BG4 peaks are found in 3′ UTRs. A possible explanation for this is that G4s within 3′ UTRs are more often unfolded or bound by proteins that mask detection by BG4 as opposed to the G4s within 5′ UTRs. The proteins that recognise G4s within 5′ UTRs may also differ from the ones that bind G4s in 3′ UTRs and may therefore differently impact recognition by BG4.

Confirming identification of G4 structures in RNA, BG4 peaks show statistical enrichment for G4 motifs (three-tetrad G4s > 3-fold; two-tetrad G4s > 2.5-fold, FDR < 0.001; Fig. 2e). BG4 uvRIP peaks were found statistically enriched within iCLAE binding sites for DHX36, DDX3X and GRSF1 when compared to random chance (> 4-log_2_fold; FDR < 0.001; Fig. 2f). BG4 uvRIP confirmed folded G4 structure at 513 iCLAE sites, many of which comprised persistent G4s that are recognised by two or more G4-interacting proteins (Fig. 2g). Many BG4 peaks (64%) did not overlap with our iCLAE datasets (Fig. 2g) and may represent G4s which are not protein-bound, though they may be partially bound by proteins not assayed in this study. It is conceivable that BG4 uvRIP requires a substantial proportion of the transcript population to be folded and therefore does not detect sites with transient G4 folding. Thus, despite the presence of RNA binding proteins in crosslinked lysates, G4 structures can be detected using a structure-specific probe, which further supports G4 formation in cellular transcripts.

### G4s in ribosomal protein mRNA

To gain insights into the biological role of RNA G4s, we focused on the 513 sites that were shown to interact with at least one of the G4 binding proteins DDX3X, DHX36 or GRSF1 in iCLAEs and also detected independently by BG4 uvRIP (Fig. 2g, Supplementary Fig. S8). Our attention was particularly drawn to the significant enrichment (Bonferroni p-value = 4.7 × 10^−8^) of such ‘BG4-confirmed’ RNA G4s within mRNA coding for ribosomal proteins, based on functional annotation analysis using DAVID (LHRI), where no other functional annotation term was found to be significantly enriched. On further scrutiny of our iCLAE data, we also found that majority of annotated ribosomal protein mRNA (61/82) contain a G4-motif within their 5′ UTR that is bound by one or more G4-binding proteins. Analysis of iCLAE sites common to all three G4-binding proteins assayed by us also showed a significant enrichment for ribosomal protein mRNAs (Fig. S9a; DAVID Bonferroni p-value = 3.2 × 10^−25^).

**Figure 3:**
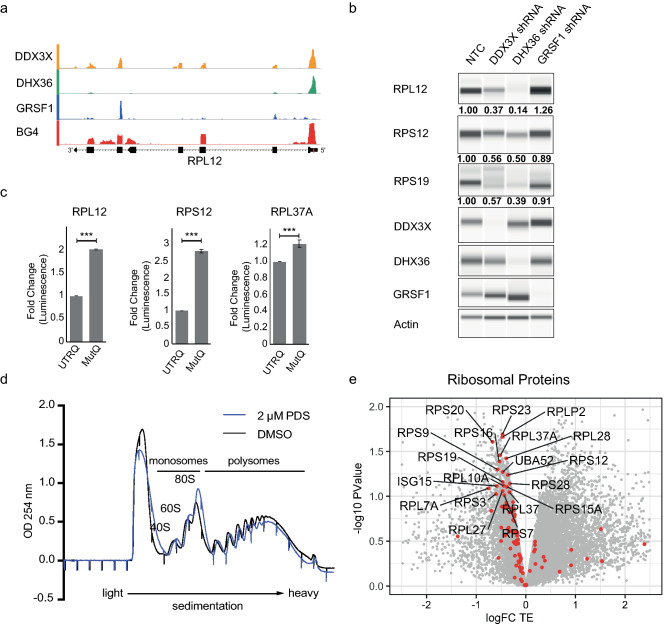
RNA G4s in ribosomal protein mRNA regulate translation. (**a**) Example snapshots demonstrating BG4 peaks in 5′ UTRs and overlapping iCLAE peaks. BG4 track depict logFC of BG4 IP vs input and iCLAE track depict counts per million. (**b**) Western blot analysis of ribosomal protein levels following shRNA mediated depletion of DDX3X, DHX36 and GRSF1. Actin serves as loading control. Number below each lane represents fold change in ribosomal protein levels compared to non-targeting control (NTC) when normalised to actin. (**c**) Fold change in luminescence comparing in vitro translation of luciferase gene from wild-type ribosomal protein UTRs containing G4 (UTRQ) to mutant UTRs with G4 disruption (MutQ). (**d**) UV absorption at 254 nM following sucrose fractionation for polysome profiling of cells treated with DMSO (black) and 2 μM PDS (blue) for 45 min. Monosomal and polysomal fractions are highlighted. (**e**) Fold change in translation efficiency (logFC TE) of each transcript plotted against the significance (− log10 p-value) over three independent biological replicates. All ribosomal protein mRNAs are highlighted in red and those with significant changes (FDR < 0.1) are labelled with the corresponding protein names.

Ribosomal protein mRNAs contain a 5′ terminal oligopyrimidine (TOP) motif which is followed by a G-rich stretch^34^ in which we identified G4 structures that show sequence conservation in higher vertebrates (Fig. 3a, Table S1). The folding of a selection of these ribosomal protein mRNA sequences into G4 structures was confirmed in vitro using a Thioflavin-T binding^35^, circular dichroism (CD) and thermal melting^36,37^ assays. G4-containing oligonucleotides bound Thioflavin T and showed increased fluorescence when compared to mutated G4 oligos containing G to A mutations that disrupt G4 formation (Fig. S10a). Ribosomal protein RNA G4s also demonstrate a CD spectrum typical of parallel G4s with a molar ellipticity maxima at 265 nM and a minima at 240 nM (Fig. S10b) with the mutation of guanines leading to the loss of this characteristic spectrum^2,22^. The thermal stability of these G4s was then assessed by measuring circular ellipticity at the 265 nM maxima in thermal melting assays (Fig. S10c). Typical of G4 folding, ribosomal protein G4 oligonucleotides had melting transition temperatures of 55–65 °C in 100 mM K^+^ and showed considerably lower melting transition temperatures under conditions that promote G4 unfolding or upon G to A mutation (Figs. S10c, S11). Collectively, these data indicate that the G4s within the 5′ UTRs of ribosomal protein mRNAs are able to physically fold into *bona fide* G4 structures under physiological salt and temperature.

### Helicases regulate ribosomal protein production

Since G4s in ribosomal protein mRNA are bound by one or more G4-interacting proteins (Fig. 3a, Supplementary Fig. S8), we assessed how the loss of these proteins impacts ribosomal protein levels. DDX3X, DHX36 and GRSF1 were each depleted using doxycycline inducible short hairpin RNAs and levels of ribosomal proteins compared to cells expressing a non-targeting control hairpin (NTC; Fig. 3b). Depletion of helicases DDX3X and DHX36 caused clear reductions in multiple ribosomal proteins that contain BG4 and in vitro confirmed G4s within the 5′ UTRs. No alterations in the respective mRNA levels were observed, indicating a translational effect (Fig. S9b). GRSF1 knockdown failed to alter ribosomal protein levels, which suggests an alternative function for GRSF1 at these transcripts. These data suggest that helicase activity of DDX3X and DHX36 is required to allow optimal ribosomal protein production. Consistent with these observations, our analysis of SILAC mass spectrometry data from DHX36 knockout cells shows that ribosomal proteins comprise the most significantly (DAVID Bonferroni p-value = 1.8 × 10^−26^) downregulated protein family^16^. Moreover, depletion of DDX3X in melanoma cells has been reported to inhibit the translation of ribosomal protein mRNAs and cause reduction in overall cellular translation^38^, however the involvement of G4s was not noted by the authors. These data show that G4-unwinding helicases regulate the expression of ribosomal proteins.

### A novel role for RNA G4s in regulating ribosomal protein synthesis

Our data show that majority of ribosomal protein mRNAs harbour a folded G4 structure within their 5′ UTR, suggesting G4s as a general feature of this mRNA family. Ribosomal proteins are translationally co-regulated^34^ and we therefore reasoned that these common structural features may be involved in regulating translation and thereby control ribosome synthesis, which in turn would globally regulate translation. To specifically assess the influence of these G4s on translation, we adopted a cell-free translation system previously used for the study of *NRAS* 5′ UTR^2^. A selection of 5′ UTRs from ribosomal protein mRNA were cloned upstream of the firefly luciferase gene along with versions carrying mutations that disrupt G4 folding. Mutation of G4s led to a significant increase in translation for all ribosomal protein UTRs studied, confirming a role for these G4 structures in regulating translation of ribosomal proteins (Fig. 3c).

To extend these findings to a cellular context, we next treated cells with a G4-stabilising small molecule to explore the impact on translation of ribosomal protein mRNA. Cells were exposed to pyridostatin (PDS—2 µM) for a brief period of 45 min, which circumvents effects from transcriptional or DNA damage responses that are associated with longer treatments^39^. The impact of G4 stabilisation on translation of individual transcripts was assessed by polysome fractionation and profiling, which measures the efficiency of translation based on the level of ribosome association with individual mRNA. RNA from polysome-associated fractions was isolated and sequenced along with total RNA from cell lysates (Fig. 3d). The translational efficiency for each transcript was calculated as the ratio of polysomal RNA to total RNA, which accounts for any change in transcript abundance upon PDS treatment. Changes in translational efficiency upon PDS treatment (log_2_FC TE) and statistical significance were determined from three independent biological replicates (Fig. 3e, Data S2). Supporting our hypothesis that ribosomal protein mRNAs represent a family of transcripts that are co-regulated by RNA G4s, functional annotation analysis of transcripts with significantly reduced translational efficiency showed notable enrichment of ribosomal protein mRNAs (DAVID Bonferroni p-value—4.8 × 10^−3^). Strikingly, most ribosomal protein mRNA showed a decrease in translation upon PDS treatment (Red dots, Fig. 3e). These effects are not due to possible DNA damage induced by PDS treatment^39^, since no increase in DNA damage markers such as γH2AX was observed with the low dose and short treatment duration used in these experiments (Fig. S12a). To determine the effects of G4-stabilisation by PDS on mature, cellular ribosomal protein levels, we next employed tandem mass tag (TMT)-based quantitative mass spectrometry (Data S3). In agreement with our polysome profiling data, we observed a decrease in levels of individual ribosomal proteins following PDS treatment (2 µM for 120 min) as seen by gene set enrichment analysis (FDR < 0.001, Normalised enrichment score = − 2.54, Fig. S12b, Data S4).

## Discussion

Overall, we have uncovered a set of cellular RNA G4s based on their recognition by a G4-structure specific antibody and endogenous G4-interacting proteins. Our work specifically highlights G4s as a feature of ribosomal protein mRNA. Appraising rG4-seq^5^ and Keth-seq^9^ datasets reveals that these methods also detected the presence of G4s in ribosomal protein mRNA. Through chemical biology interventions we have discovered that stabilised G4s interfere with translation of ribosomal proteins, which also explains the reduced ribosomal protein synthesis that is seen following loss of RNA G4 resolving proteins such as DDX3X and DHX36. Through inspection of publicly available data, we found ribosomal protein mRNA are also targets of CNBP (cellular nuclear acid binding protein). CNBP has been suggested to bind G-rich regions to prevent G4 formation thus facilitating translation^40^. Our scrutiny of proteomics data following depletion of CNBP also reveals a reduction in ribosomal protein levels^40^. Together with observations in this study, this points to multiple G4-interacting proteins with a role in regulating ribosomal protein synthesis.

It is unclear if these proteins can co-occupy the same individual transcript or whether they interact with distinct transcript sub-populations from the same gene. Another unresolved question is why the loss of individual G4 resolving proteins is not compensated by the activity of others that seem to engage the same target G4, including the ubiquitous helicase EIF4A? One possible explanation may be that the loss one of these proteins shifts the balance resulting in sequestration into sub-cellular compartments such as stress granules to modulate translation. Stress granules are cytoplasmic protein-RNA aggregates that form in response to cell stress and mRNA with more structured UTRs show greater propensity to localise to these bodies^41^. Depletion of DDX3X and DHX36 has been reported to cause increased formation of stress granules^16,42^.

Cellular ribosomal protein levels influence global translation output^34^. Our polysome profiling data with 2 µM PDS showed a small decrease of RNA in the polysomal fractions indicating an impact on global translation (Fig. 3d). When cells are treated with a higher concentration of PDS (10 µM for 45 min), we observe a larger reduction in overall cellular translation as seen by reduced absorbance in the polysomal fractions and corresponding increase in monosomal fractions (Fig. S12c). Collectively, our data suggest that conserved RNA G4s in ribosomal protein mRNA may function to regulate global translational rates.

Protein synthesis is unquestionably essential for cellular growth and survival and thus is exquisitely regulated. Ribosome biogenesis is tightly co-ordinated so that equimolar quantities of protein and RNA components are synthesised^34^. As ribosomal protein levels are translationally coregulated, we propose that ribosome production is modulated by G4 formation in ribosomal protein mRNA. Our findings unveil a novel mechanism that regulates synthesis of cellular translational apparatus and underline a potential therapeutic avenue for intervention in diseases with dysregulated translation, such as cancer.

## Methods

### Cell culture

Inducible Flp-In T-REx 293 (ThermoFisher) stable cell lines expressing Strep-tag (ST)/HA affinity tagged GRSF1 and DHX36 were generated by transfection of pcDNA5/FRT/TO vector as per manufacturers protocol (ThermoFisher). Cells were selected with Blasticidin (Gibco) and Hygromycin D (Sigma). For shRNA knockdowns, SMARTvector inducible lentiviral shRNA system (Dharmacon) was used to generate viral particles. Transduction efficiency was optimised to 30% to ensure single incorporation events and hairpin expressing cells selected as per manufacturers’ instructions. Protein knockdowns were achieved by treating cells with 1 μg/ml Doxycycline for 72 h.

### Western blotting

Cells were lysed in Pierce™ RIPA buffer (Thermo Fisher) as per manufacturer’s instructions. Protein lysates were quantified using Direct Detect^®^ infrared spectrometer (Merck Millipore), equal amounts of lysate subjected to SDS PAGE and immunoblotting, and signal was visualised and quantified using Odyssey CLx and Image Studio (Licor) using the following antibodies actin (4970S; Cell Signaling Technology) and γH2AX (ab11174; Abcam). WES (ProteinSimple) automated Western Blotter was used with antibodies against RPL12 (14536-1-AP; Proteintech), RPS12 (16490-1-AP; Proteintech), RPS19 (15085-1-AP; Proteintech), DHX36 (ab194358; abcam); DDX3X (sc-365768; Santa Cruz Biotech.); GRSF1 (ab194358; Abcam) and actin (a5441; Merck and #4970; Cell Signalling Tech.).

*ELISA* assays using recombinant proteins were performed as in Zyner et al.^43^, using the NRAS G4 oligo 5′-UGU GGG AGG GGC GGG UCU GGG UGC-3′ and NRAS mG4 oligo 5′-UGU AGA AAG AGC AGA UCU AGA UGC-3′. Purified recombinant DDX3X was obtained from OriGene Technologies (TP304171) and recombinant DHX36 was purified as described in Chen^13^.

### In vitro translation

5′ UTRs from ribosomal protein mRNA preceded by a minimal T7 promoter and a *Hind*III restriction site, and followed by 5′ section of the firefly luciferase gene and *BstB*I restriction site were ordered as gene blocks (ThermoFisher). Restriction digested gene blocks were used to replace the NRAS 5′ UTR in the pUC18 vector described in Kumari et al.^2^. G4 sequences were mutated by site directed mutagenesis. In vitro translations assays were performed as described in Kumari et al.^2^. Briefly, linearised plasmids were in vitro transcribed using the mMessage mMachine^TM^ T7 transcription kit (ThermoFisher) as per manufacturers’ instructions. Resulting capped mRNA were gel purified with Zymoclean™ RNA gel recovery kit (Zymo Research). Equimolar amounts of UTRQ and mutQ RNA were in vitro translated using nuclease treated Rabbit Reticulocyte lysates (Promega) as per manufacturers’ instructions. Luciferase activity was determined with Steady-Glo^®^ Luciferase assay system (Promega) and luminescence measure with a CLARIOstar microplate reader (BMG Labtech).

*iCLAE* experiments were performed as in Herdy et al.^11^. Briefly, protein expression was induced using 0.01 μg/ml Doxycycline overnight. Cells were UV-crosslinked (254 nM, 200 mJ/cm^2^) and cytoplasmic lysates collected by hypotonic lysis. Lysates were adjusted to a final concentration of 50 mM Tris–HCl (pH 7.4) 100 mM NaCl and 0.1% SDS, and subjected to limited RNase digestion for 3 min at 37 °C. Lysates were subjected to affinity enrichment and iCLAE library prep as described in Herdy et al.^11^ and Huppertz et al.^29^. Reverse transcription was performed with SuperScript III using G4 optimised lithium buffers as in Kwok et al.^5^.

### BG4 uvRIP

The protocol was adapted from G4 ChIP as in Hansel-Hertsch et al.^44^. Crosslinked cytoplasmic lysates were generated using hypotonic lysis as for iCLAEs. RNA concentration was quantified using Qubit broad range RNA assay kits. For each biological replicate, seven 1.5 ml tubes containing 43 μl of blocking buffer (25 mM HEPES pH 7.5, 10.5 mM NaCl, 110 mM KCl, 1 mM MgCl_2_ and 1% (w/v) BSA) and 2.5 μl of lysates measuring 400–600 ng/μl RNA were prepared on ice. All aliquots were digested with 2U Turbo™ DNase (Thermo Fisher) at 37 °C for 5 min and cooled on ice. Limited RNaseA digestion was performed at 37 °C for 3 min, aliquots rapidly cooled on ice and 1 μl RNasin^®^ (Promega) added to each aliquot. The remainder of the protocol was performed on ice.

2.5 μl Flag-tagged BG4 antibody (4 μM) or 2.5 μl A9 antibody (μM) were added to three aliquots each and 2.5 μl PBS was added to one aliquot that was used as Input and left at 4 °C until the proteinase K digestion step. All aliquots were incubated at 4 °C for 60 min. Anti-FLAG M2 magnetic beads (65 μl, Sigma) were washed three times with block buffer and resuspended in 650 μl block buffer. 50 μl beads were added to each BG4 and A9 IP aliquots, and all aliquots incubated at 4 °C for a further 60 min. The BG4 and A9 IPs were then washed with 200 μl wash buffer (100 mM KCl, 0.1% Tween20, 10mM TRIS pH 7.4) four times at 4 °C and twice for 10 min shaking at 37 °C. The IP aliquots were resuspended in 150 μl PK-SDS buffer (100 mM Tris pH 7.4, 50 mM NaCl, 1 mM EDTA, 0.2% SDS) and pooled into one BG4 and A9 sample respectively. 5 μl of the Input was added to 445 μl PK-SDS buffer to give a 10% Input. Samples were de-crosslinked and de-proteinated by addition 10 μl Proteinase K (20 mg/ml, Thermo Fisher) and incubation at 50 °C for 60 min. RNA was purified using Phenol/Chloroform/isoamyl alcohol, phase separated by Heavy Phase lock tubes (QuantaBio) and precipitated overnight at − 20 °C with 1.5-fold volume of isopropanol, 150 mM sodium acetate pH 5.5 and 1 μl GlycoBlue™ (Thermo Fisher). Samples were centrifuged for 30 min at 4 °C and RNA pellet resuspended in 15 μl water.

RNA subjected to Ribodepletion with NEBNext^®^ rRNA depletion kit (New England Biolabs) as per manufacturer’s instructions with one alteration that allows for purification of small RNA fragments. Ribodepleted RNA was not purified with NEBNext^®^ RNA sample purification beads as described by the manufacturer but with phenol chloroform purification as described above. Libraries were then prepared using NEBNext^®^ Ultra™ II Directional RNA Library Prep Kit (New England Biolabs) as per manufacturer’s protocol with the following alterations. Quadruplex optimised reverse transcription buffer^5^ was used for first strand synthesis instead of manufacturer supplied buffer. Following second strand synthesis, cDNA was purified using Qiaquick^®^ nucleotide removal kit (Qiagen) instead of using SPRISelect beads (Beckman Coulter™).

### iCLAE and BG4 uvRIP data analysis

iCLAE and BG4 uvRIP sequencing data were processed and peaks were called as described in https://github.com/sblab-bioinformatics/rna-g4-proteins. The previously published DDX3X libraries were reprocessed using this approach for consistency. Briefly, libraries were demultiplexed, filtered based on quality, duplicates removed and aligned to the human reference genome (GRCh38, GENCODE release 28) using bwa allowing for 0.06 err rate^45^.

For iCLAE, aligned reads were further deduplicated using unique barcode identifiers within the adapters. Peaks were called using Piranha^46^ using a p-value cutoff of 0.0001 and consensus peaks defined as peaks occurring in two or more biological replicates. For BG4 uvRIP, all regions of read density above 1 count per million in the BG4 and A9 libraries were identified and reads aligned within these regions counted for all input and IP libraries. Peaks were defined as regions of differential enrichment compared to input (log_2_FC > 0.8 and FDR < 0.05) using differential binding analysis in EdgeR^47^. Overlaps and enrichments over genomic features and G4 motifs were obtained using bedtools^48^ and the genomic association tester (GAT^49^). Functional annotation analysis was performed using DAVID (LHRI) and GSEA (Broad Institute). Tracks shown in the manuscript comprise BigWig files generated by aggregation of individual biological replicates. Where indicated BG4 uvRIP tracks show BigWig files generated by calculating the log_2_FC between cumulative BG4 or A9 tracks to the cumulative input track.

### Polysome profiling and sequencing

Polysome profiles were prepared as previously described^50^. Briefly, cells treated for 45 min with indicated concentration of PDS or DMSO were incubated with cycloheximide, lysed and polysome fractions collected. Total RNA and RNA from polysomal fractions was isolated using TRIzol^®^ (Life Technologies) as per manufacturer’s instructions. Library preparation was performed using the TrueSeq RNA library prep kit (Illumina). Translational efficiency was calculated as the ratio of counts per million in polysome fractions to counts per million in total lysate. Detailed data analysis pipeline was as described on https://github.com/sblab-bioinformatics/rna-g4-proteins.

### Thioflavin-T binding, circular dichroism and thermal melting

RNA oligonucleotides purchased from IDT were diluted to 10 μM in 10 mM Lithium cacodylate buffer (pH 7.4) with 150 mM LiCl or KCl and were folded by heating at 95 °C for 5 min and slow cooling to 4 °C at the rate of 0.5 °C/min. Thioflavin-T binding assay was performed as described in Xu et al.^35^. CD spectra were obtained on an Applied Photophysics Chirascan Plus circular dichroism spectropolarimeter.

### Sample dissolution, TMT labelling and reverse-phase fractionation

Cells were treated with 2 μM PDS for two hours and harvested in ice cold PBS containing protease and phosphatase inhibitors (Roche). Cell pellets resuspended in lysis buffer containing 100 mM Triethylammonium bicarbonate (TEAB, Sigma), 0.1% SDS were heated at 90 °C for 5 min and sonicated. Protein concentration was estimated using Bradford assay (BIO-RAD-Quick start). 90 μg of total protein were reduced with 2 μl of 50mM tris-2-caraboxymethyl phosphine (TCEP, Sigma) for 1 h at 60 °C followed by alkylation with 1 ul of 200mM methyl methanethiosulfonate (MMTS, Sigma) for 10 min at room temperature (RT). Then protein samples were digested overnight at 37 °C using trypsin solution at ratio protein/trypsin ~ 1:30. The next day, protein digest was labelled with the TMT-10plex reagents (Thermo Scientific) for 1 h. The reaction was quenched with 8 μl of 5% hydroxylamine (Thermo Scientific) at room temperature for 15 min. All the samples were mixed and dried with speed vac concentrator. The dry TMT mix was fractionated on a Dionex Ultimate 3000 system at high pH using the X-Bridge C18 column (3.5 μm, 2.1 × 150 mm, Waters) with 90 min linear gradient from 5 to 95% acetonitrile contained 20 mM ammonium hydroxide at a flow rate of 0.2 ml/min. Peptides fractions were collected between 20 and 55 min and were dried with speed vac concentrator. Each fraction was reconstituted in 0.1% formic acid for liquid chromatography tandem mass spectrometry (LC–MS/MS) analysis.

### LC-MS/MS and data processing

LC-MS/MS was performed as in Papachristou et al.^51^. Briefly, peptide fractions were analysed on a Dionex Ultimate 3000 system coupled with the nano-ESI source Fusion Lumos Orbitrap Mass Spectrometer (Thermo Scientific). Peptides were trapped on a 100 μm ID × 2 cm microcapillary C18 column (5 µm, 100A) followed by 2 h elution using 75 μm ID × 25 cm C18 RP column (3 µm, 100 Å) at 300 nl/min flow rate. In each data collection cycle, one full MS scan (380–1500 m/z) was acquired in the Orbitrap (120 K resolution, automatic gain control (AGC) setting of 3 × 10^5^ and Maximum Injection Time (MIT) of 100 ms). The subsequent MS2 was conducted with a top speed approach using a 3-s duration. The most abundant ions were selected for fragmentation by collision-induced dissociation (CID). CID was performed with a collision energy of 35%, an AGC setting of 1 × 10^4^, an isolation window of 0.7 Da, a MIT of 35 ms. Previously analysed precursor ions were dynamically excluded for 45 s. During the MS3 analyses for TMT quantification, precursor ion selection was based on the previous MS2 scan and isolated using a 2.0 Da m/z window. MS2–MS3 was conducted using sequential precursor selection (SPS) methodology with the top10 settings. HCD was used for the MS3, it was performed using 55% collision energy and reporter ions were detected using the Orbitrap (50K resolution, an AGC setting of 5 × 10^4^ and MIT of 86 ms).

The Proteome Discoverer 2.1. (Thermo Scientific) was used for the processing of CID tandem mass spectra. The SequestHT search engine was used and all the spectra searched against the Uniprot Homo sapiens FASTA database (taxon ID 9606—Version February 2017). All searches were performed using as a static modification TMT6plex (+ 229.163 Da) at any N-terminus and lysines and Methylthio at Cysteines (+ 45.988 Da). Methionine oxidation (+ 15.9949 Da) and Deamidation on Asparagine and Glutamine (+ 0.984) were included as dynamic modifications. Mass spectra were searched using precursor ion tolerance 20 ppm and fragment ion tolerance 0.5 Da. For peptide confidence, 1% FDR was applied and peptides uniquely matched to a protein were used for quantification. Differential analysis was performed as described on https://github.com/sblab-bioinformatics/rna-g4-proteins.

## Supplementary Information

Supplementary Information 1.

Supplementary Information 2.

Supplementary Information 3.

## Data Availability

Sequencing data are available at gene expression omnibus reference GSE154570 and scripts used to analyse the data are accessible at https://github.com/sblab-bioinformatics/rna-g4-proteins.
