# Supplementary material for: RNA G-quadruplex structures control ribosomal protein production
Source: Sci Rep. 2021 Nov 23;11:22735. doi: 10.1038/s41598-021-01847-6 (PMC8611094; doi:10.1038/s41598-021-01847-6)

Supplementary Information: Blots and Gels

RNA G-quadruplex structures control ribosomal protein production

Dhaval Varshney[1], Sergio Martinez Cuesta[1,4], Barbara Herdy[1], Ummi Binti Abdullah[1,5], David Tannahill[1], Shankar Balasubramanian[1,2,3]*

[1]Cancer Research UK Cambridge Institute, Li Ka Shing Centre, Robinson Way, Cambridge, CB2 0RE, UK.

[2]Yusuf Hamied Department of Chemistry, University of Cambridge, Cambridge, CB2 1EW, UK

[3]School of Clinical Medicine, University of Cambridge, Cambridge, CB2 0SP, UK

[4]Present Address: Data Sciences and Quantitative Biology, Discovery Sciences, AstraZeneca, Cambridge, UK

[5]Present Address: Weatherall Institute of Molecular Medicine, University of Oxford, Oxford, UK.

*Correspondence: sb10031@cam.ac.uk (S.B.)

Figure 3b

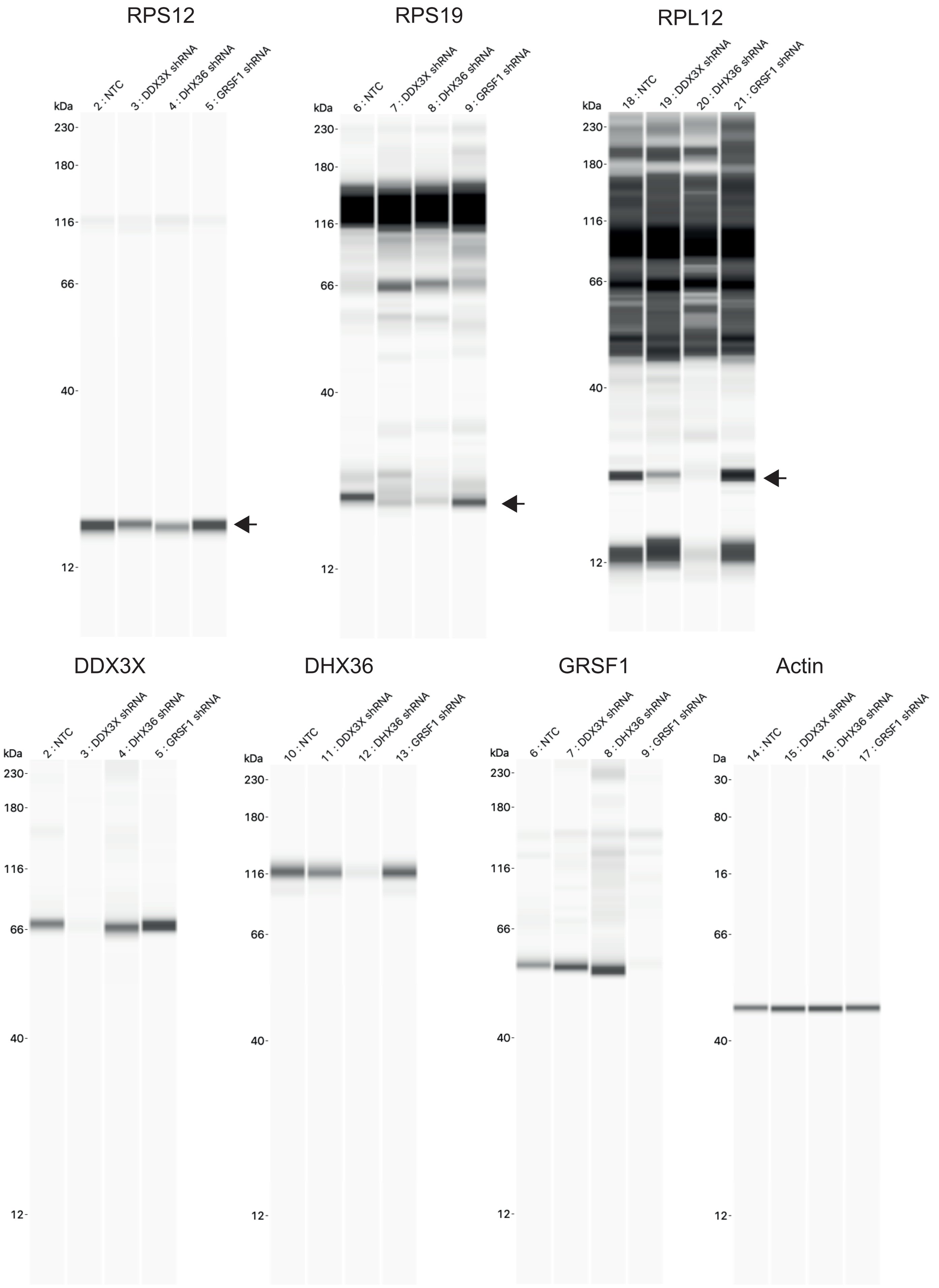

RPS12
kDa
2 : NTC
3 : DDX3X shRNA
4 : DHX36 shRNA
5 : GRSF1 shRNA
230
180
116
66
40
12
RPS19
kDa
6 : NTC
7 : DDX3X shRNA
8 : DHX36 shRNA
9 : GRSF1 shRNA
230
180
116
66
40
12
RPL12
kDa
18 : NTC
19 : DDX3X shRNA
20 : DHX36 shRNA
21 : GRSF1 shRNA
230
180
116
66
40
12
DDX3X
kDa
2 : NTC
3 : DDX3X shRNA
4 : DHX36 shRNA
5 : GRSF1 shRNA
230
180
116
66
40
12
DHX36
kDa
10 : NTC
11 : DDX3X shRNA
12 : DHX36 shRNA
13 : GRSF1 shRNA
230
180
116
66
40
12
GRSF1
kDa
6 : NTC
7 : DDX3X shRNA
8 : DHX36 shRNA
9 : GRSF1 shRNA
230
180
116
66
40
12
Actin
Da
14 : NTC
15 : DDX3X shRNA
16 : DHX36 shRNA
17 : GRSF1 shRNA
30
80
16
66
40
12

Supplementary Figure 1a

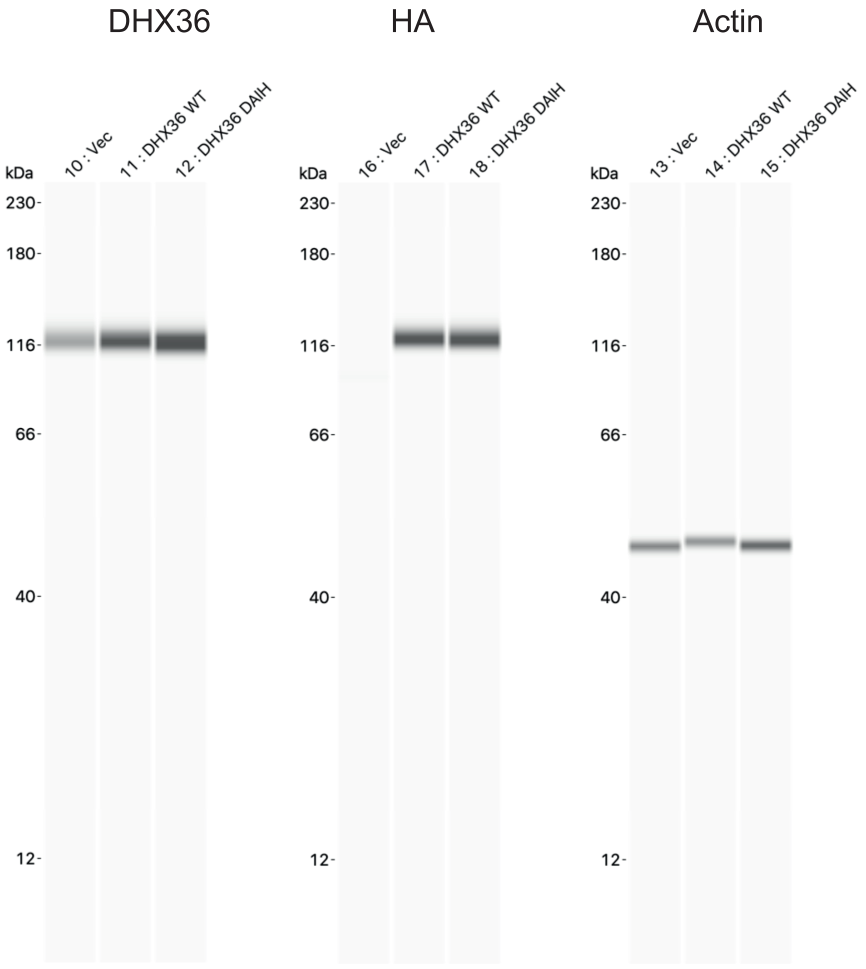

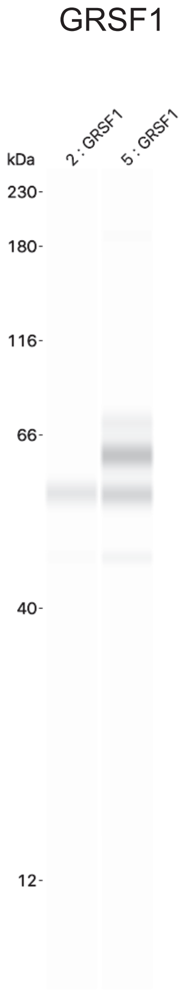

Supplementary Figure 2d

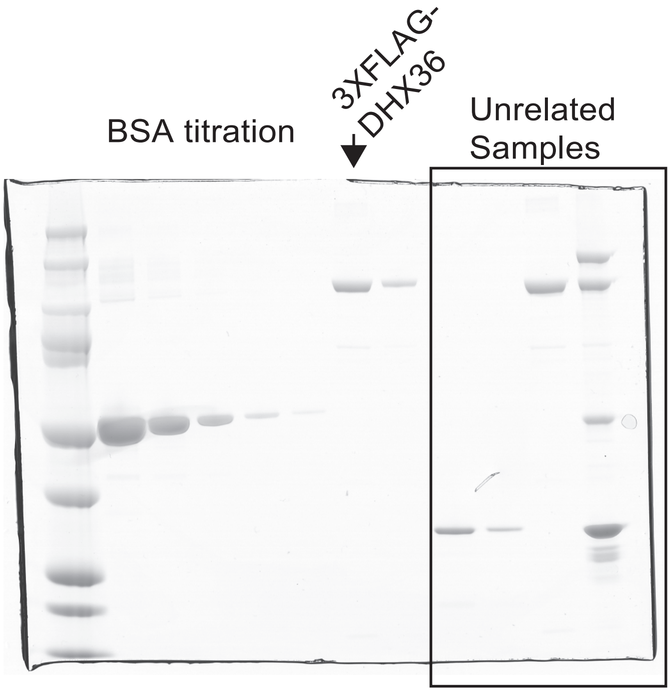

Supplementary Figure 12a

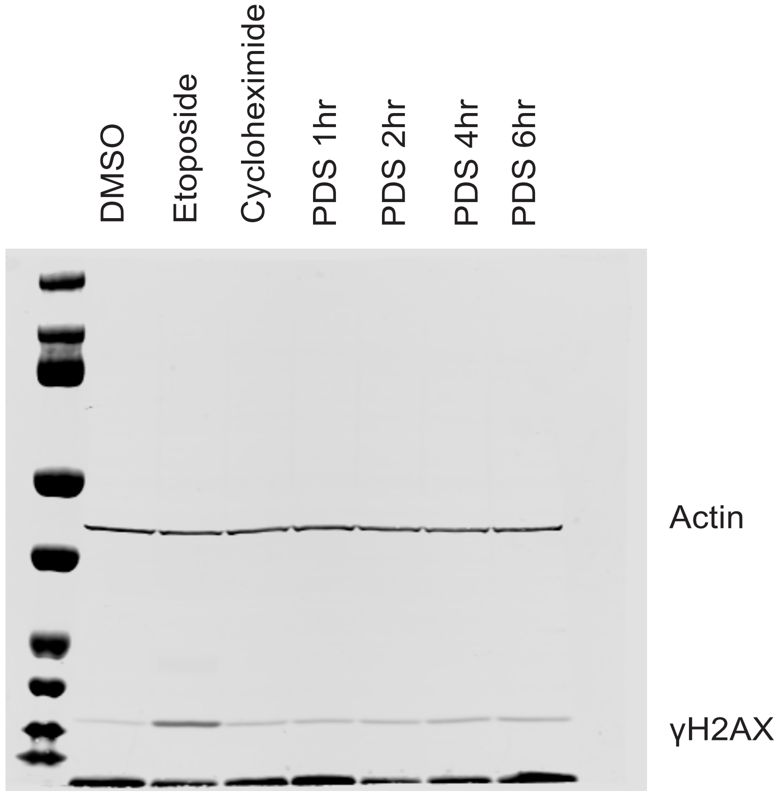

Supplement: Supplementary file 2 — Supplementary Information 2. [file 41598_2021_1847_MOESM2_ESM.pdf]
