# Supplementary material for: RNA G-quadruplex structures control ribosomal protein production
Source: Sci Rep. 2021 Nov 23;11:22735. doi: 10.1038/s41598-021-01847-6 (PMC8611094; doi:10.1038/s41598-021-01847-6)
Supplement: Supplementary file 3 — Supplementary Information 3. [file 41598_2021_1847_MOESM3_ESM.pdf]

Supplementary Information for

RNA G-quadruplex structures control ribosomal protein production

Dhaval Varshney[1], Sergio Martinez Cuesta[1,4], Barbara Herdy[1], Ummi Binti Abdullah[1,5], David Tannahill[1], Shankar Balasubramanian[1,2,3]*

[1]Cancer Research UK Cambridge Institute, Li Ka Shing Centre, Robinson Way, Cambridge, CB2 0RE, UK.

[2]Yusuf Hamied Department of Chemistry, University of Cambridge, Cambridge, CB2 1EW, UK

[3]School of Clinical Medicine, University of Cambridge, Cambridge, CB2 0SP, UK

[4]Present Address: Data Sciences and Quantitative Biology, Discovery Sciences, AstraZeneca, Cambridge, UK

[5]Present Address: Weatherall Institute of Molecular Medicine, University of Oxford, Oxford, UK.

*Correspondence: sb10031@cam.ac.uk (S.B.)

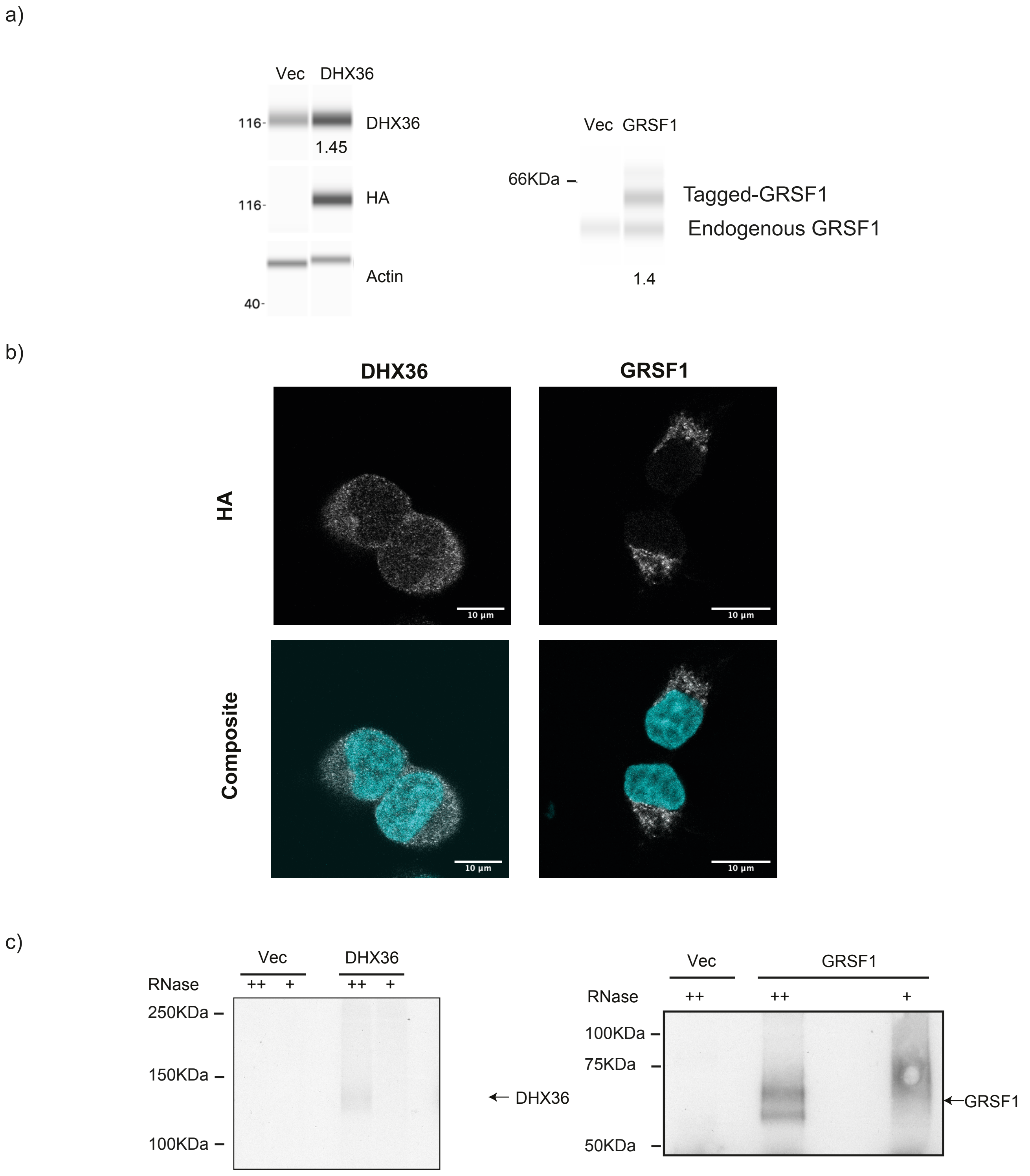

Figure S1: Expression of exogenous DHX36 and GRSF1. a) Western blots on lysates from cells expressing empty vector control (Vec), DHX36 and GRSF1. Numbers at the bottom represent fold increase in protein levels compared to endogenous (DHX36 normalised to Actin) b) Subcellular localisation visualised using immunofluorescence imaging where DAPI (shown in cyan) stains nuclear DNA and anti-HA antibody (shown in grey) stains the tagged-DHX36 and GRSF1. Scale bars measure 10 microns. c) Autoradiographs visualising radiolabelled RNA crosslinked to tagged-DHX36 or GRSF1 for iCLAE experiments. High RNase treatment (++) was used to determine if the detected RNP resolved at correct molecular weight. Low RNase treatment (+) was used to obtain a smear and isolating RNA fragments for library preparation.

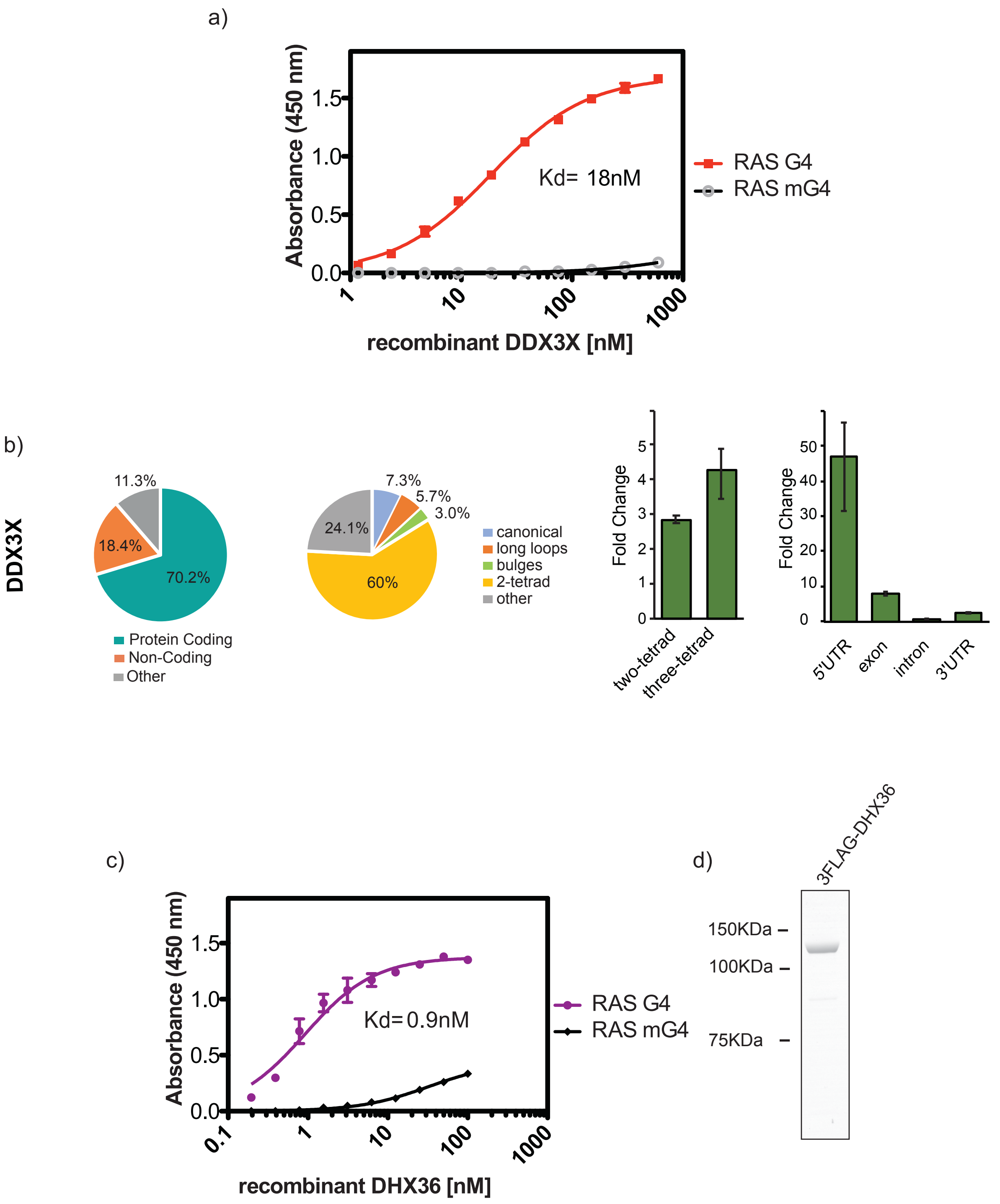

Figure S2: DDX3X and DHX36 bind RNA G4s. a,c) ELISA assay performed using NRAS G4 oligo (5′-Biotin-UGUGGGAGGGGCGGGUCUGGGUGC-3′) and mutated mG4 oligo (5′-Biotin-UGUAGAAAGAGCAGAUCUAGAUG-3′) for recombinant FLAG-tagged DDX3X (Catalogue Number: TP304171, Origene Technologies, USA) and DHX36 (expressed in house; panel d). b) Genomic localisation and motif analysis of previously published DDX3X iCLIP dataset (GSE106476). G4 motifs include the canonical three tetrad G4 motif, three tetrad G4 motif containing long loops or a single bulge or two-tetrad G4 motif

a)

b)

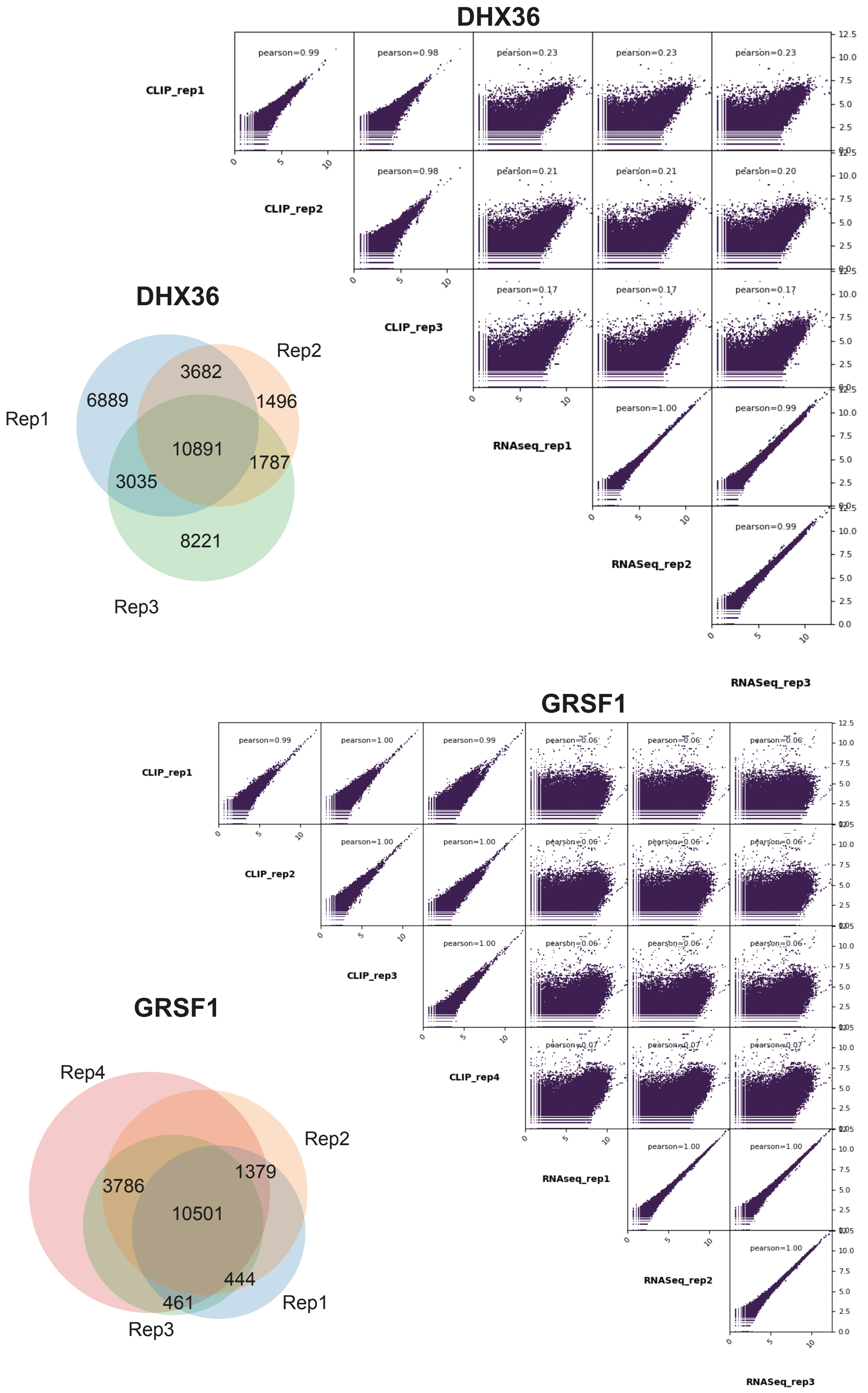

Figure S3: Correlation between individual iCLAE replicates and RNA-seq for DHX36 (a) and GRSF1 (b). Venn diagram demonstrates the overlap between the peaks for individual replicates.

a)

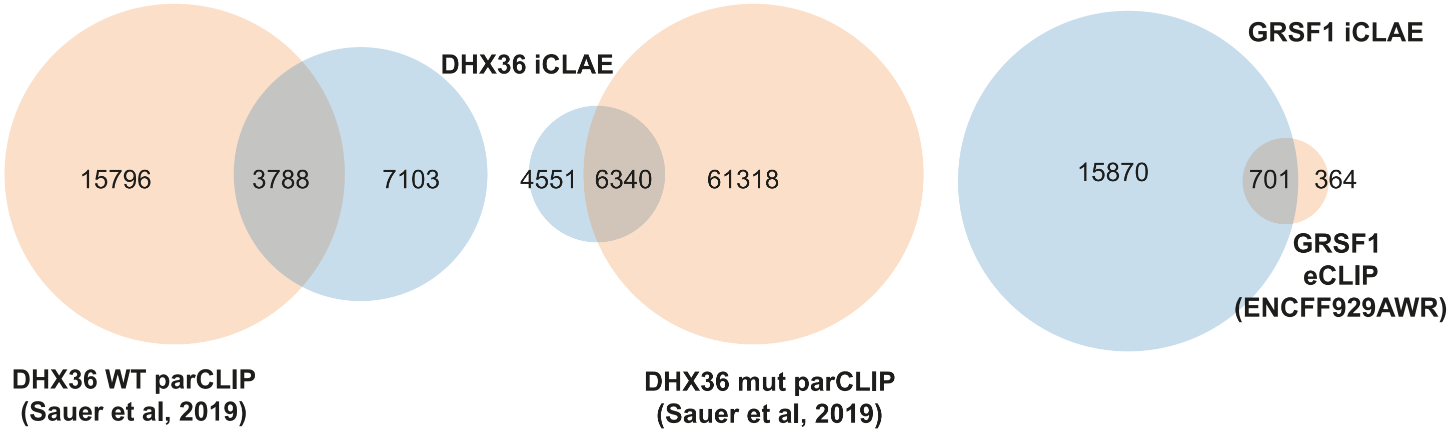

Overlap with Published Datasets					
Libraries	Peak count	Overlap	Fold Enrichment over random	CI95 Low	CI95 High
DHX36	10891				
DHX36 WT parCLIP	19584	3788	23.86	43.18	22.79
DHX36 mut parCLIP	67658	6340	53.3	58.61	48.67
GRSF1	16571				
ENCFF929AWR	1065	701	80.12	108.89	59.25

b)

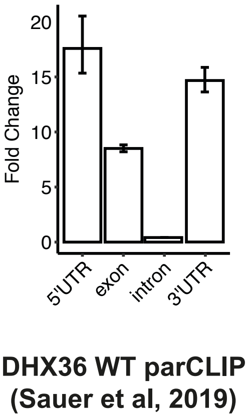

Figure S4: Overlaps of DHX36 and GRSF1 iCLAE data (blue) with published datasets. Fold enrichment of consensus peaks over published data when compared to random is represented in table below. b) Enrichment analysis depicting genomic distribution of DHX36 WT parCLIP peaks (Sauer et al, Nat. Comms., 2019).

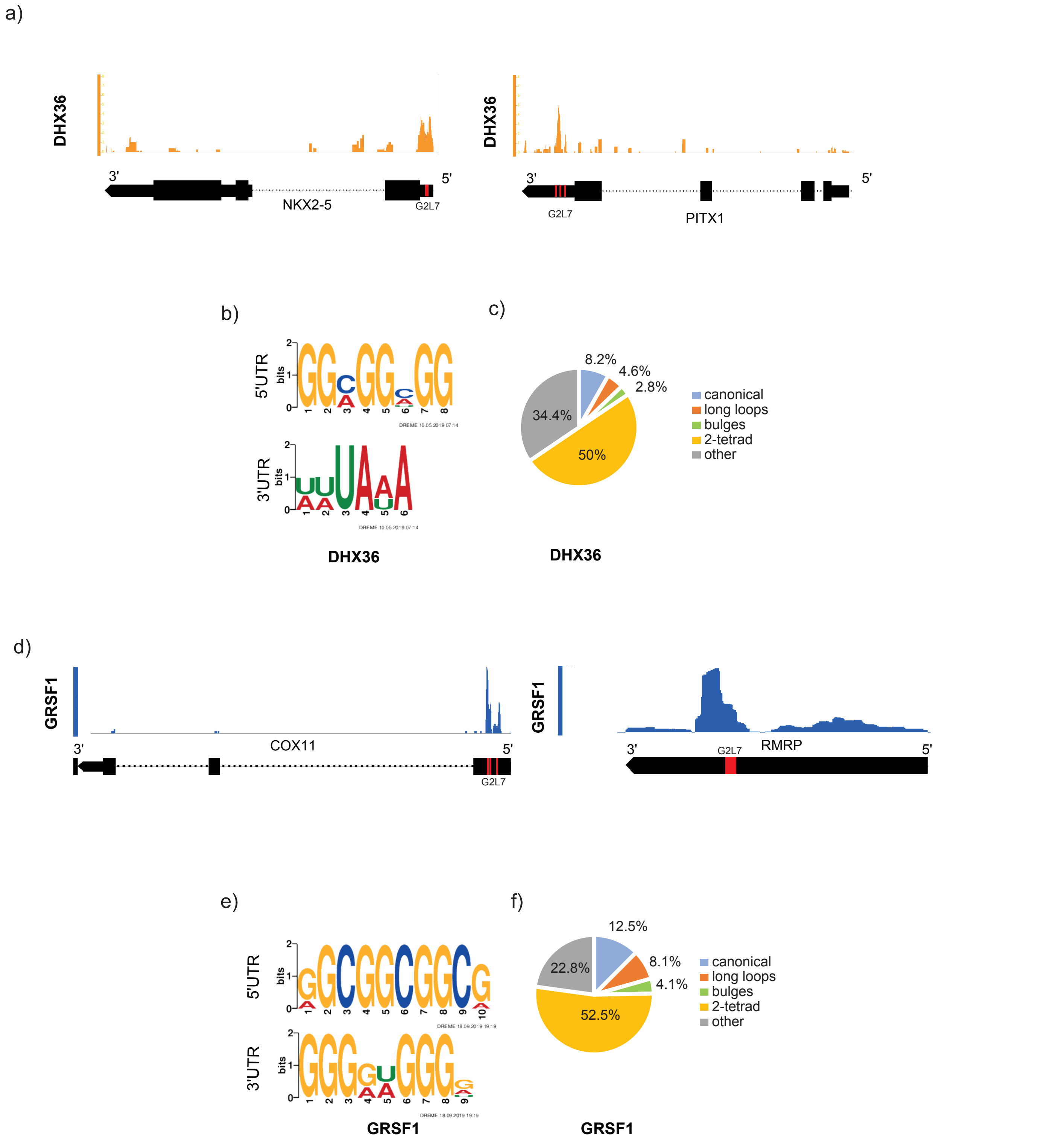

Figure S5: iCLAE for DHX36 and GRSF1. a,d) Example snapshot of DHX36 and GRSF1 iCLAE peaks on previously validated targets. Tracks normalised to counts per million. b,e) DREME motif analysis of DHX36 and GRSF1 iCLAE sites within 5′ and 3′ UTRs. c,f) Percentage overlap of DHX36 and GRSF1 iCLAE peaks with canonical three tetrad G4 motifs, three tetrad G4 motifs containing long loops or a single bulge or two-tetrad G4 motifs.

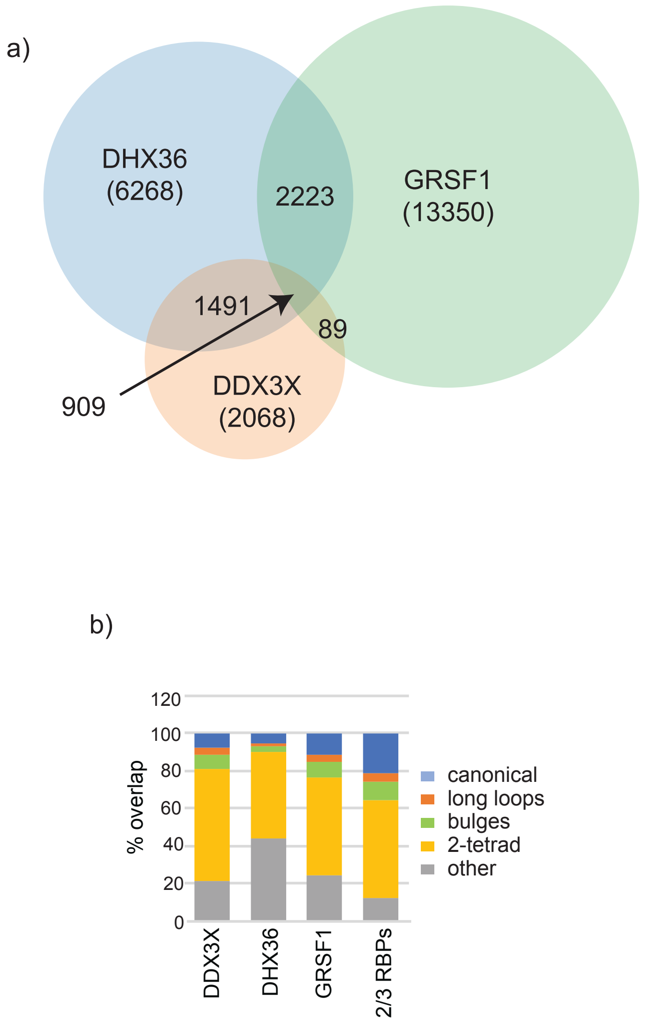

Figure S6: Overlaped binding sites for DDX3X, DHX36 and GRSF1. a) Overlap between iCLAE peaks. DDX3X dataset has been published previously (Herdy et al, Nucl. Acids Res., 2018). b) Overlap of peaks unique to individual proteins and peaks common to two or more proteins with G4 motifs.

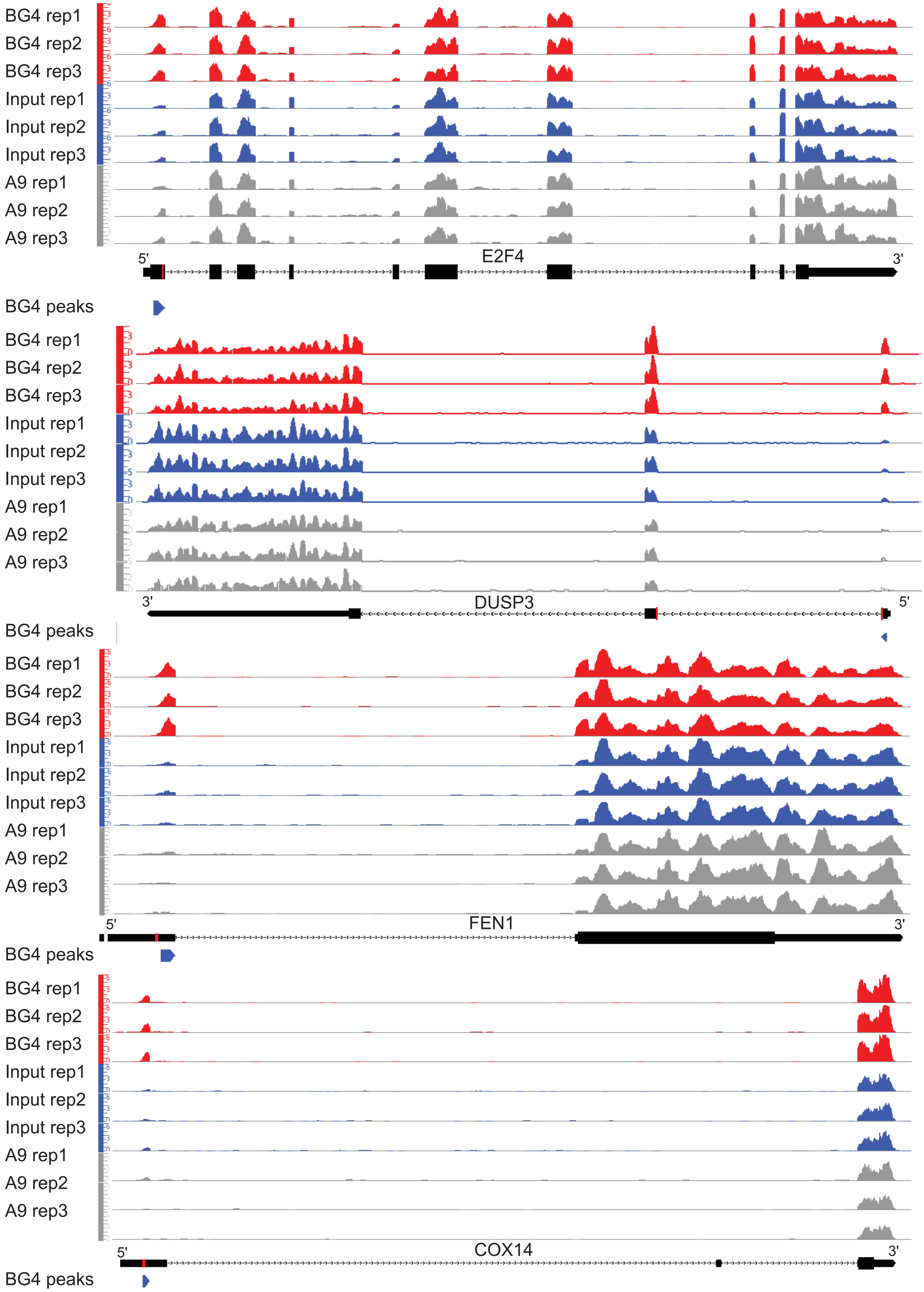

Figure S7: Examples of BG4 uvRIP peaks in 5' UTRs of protein coding genes. BG4 (red) and A9 (grey; negative control) uvRIP tracks shown alongside input (blue) signal. Tracks normalised to counts per million and shown at equal scales. Differentially enriched BG4 peaks denoted as blue arrows, where direction of the arrow depicts strand information. G4 motif associated with each peak denoted as red box in gene structure track.

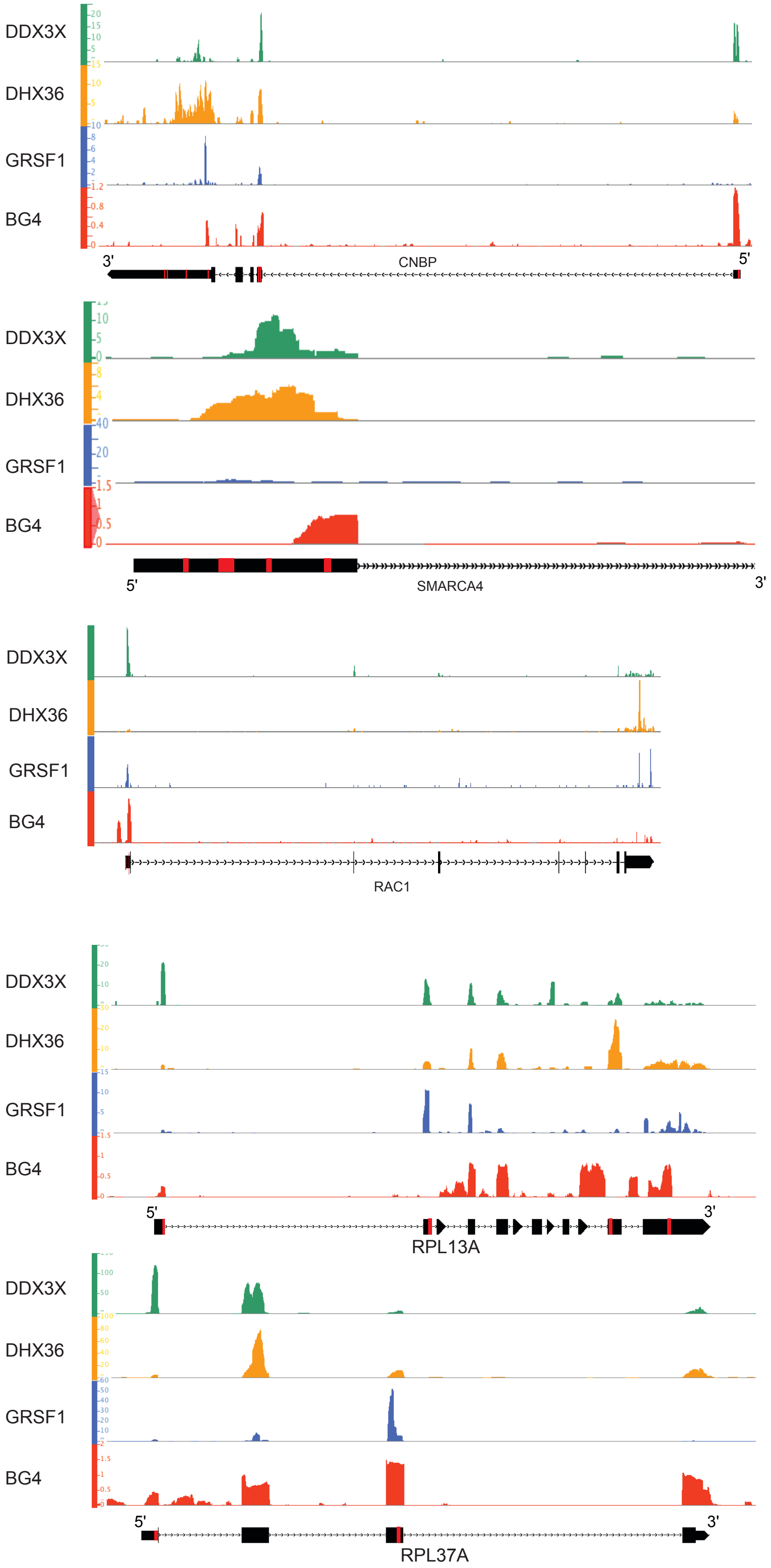

Figure S8: BG4 uvRIP and RBP iCLAE signal in 5' UTRs. BG4 track depicts logFC of BG4 uvRIP versus input. iCLAE tracks depict counts per million. G4 motifs are denoted by red boxes in gene structure track.

a)

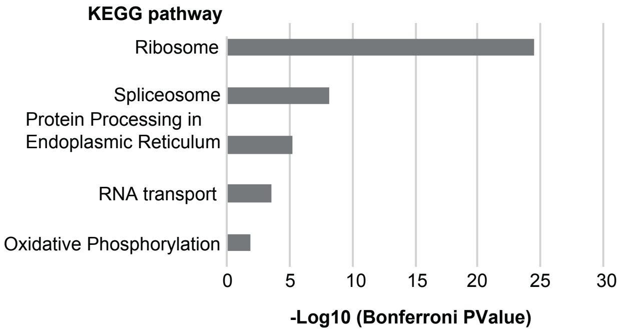

b)

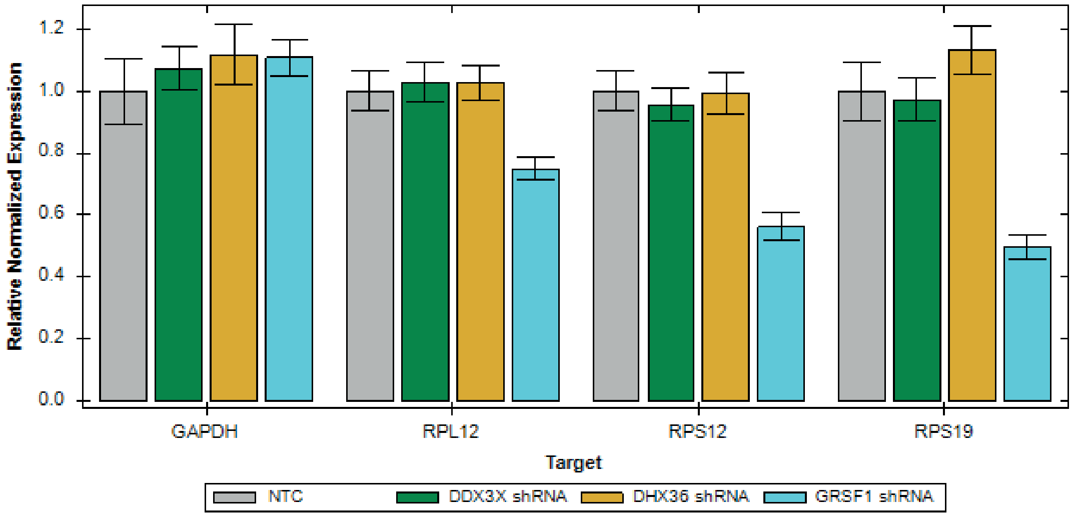

Figure S9: G4s in ribosomal protein mRNAs. a) Functional annotation analysis performed on mRNA with overlapping iCLAE peaks for DDX3X, DHX36 and GRSF1 using DAVID (LHRI). b) RT-qPCR of ribosomal protein mRNA following shRNA mediated depletion of G4-binding protein DDX3X, DHX36 and GRSF1. GAPDH provides control. Expression normalised to ARPP P0.

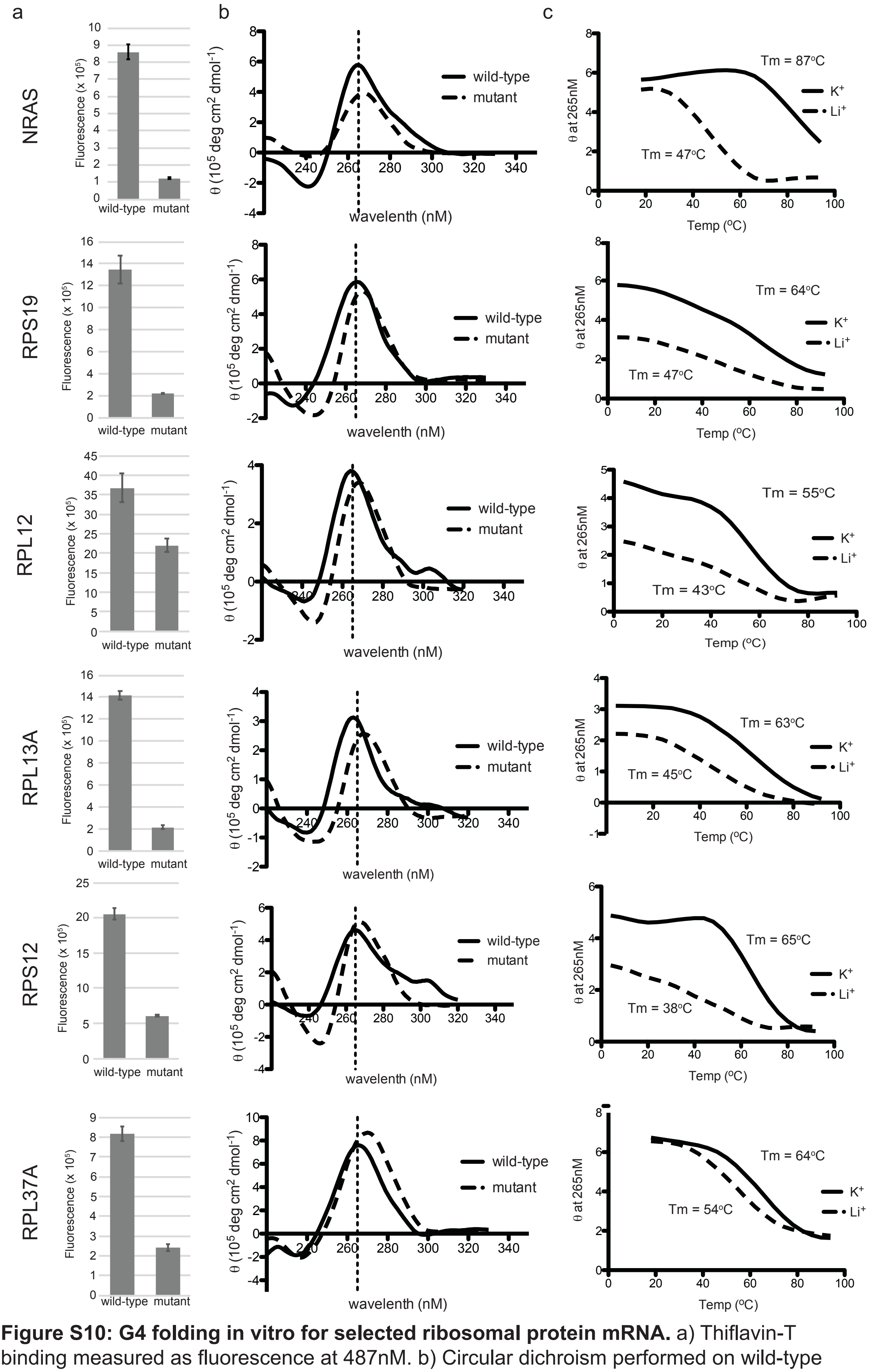

Figure S10: G4 folding in vitro for selected ribosomal protein mRNA. a) Thiflavin-T binding measured as fluorescence at 487nM. b) Circular dichroism performed on wild-type and mutant oligos for RNA G4s identified using iCLAE and BG4 uvRIP. c) Melting profiles of oligos measured at 265nM circular ellipticity maxima.

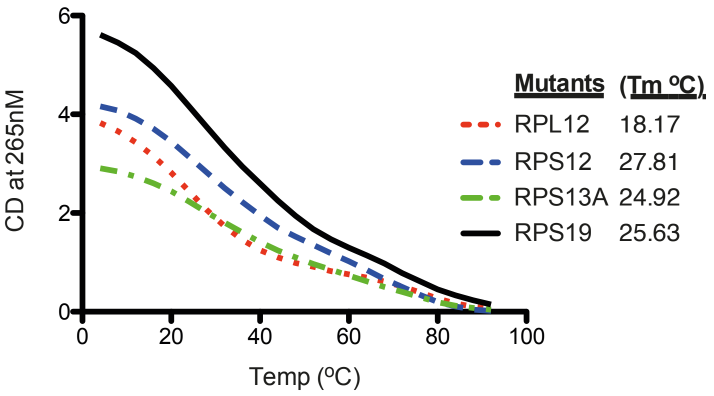

Figure S11: G to A mutations disrupt G4 formation. Melting profiles of the indicated mutated RNA oligonucleotides.

a)

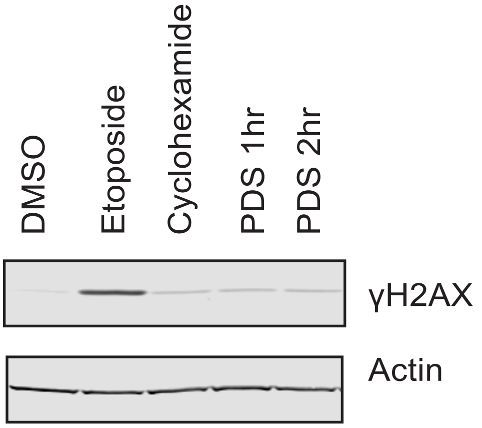

b)

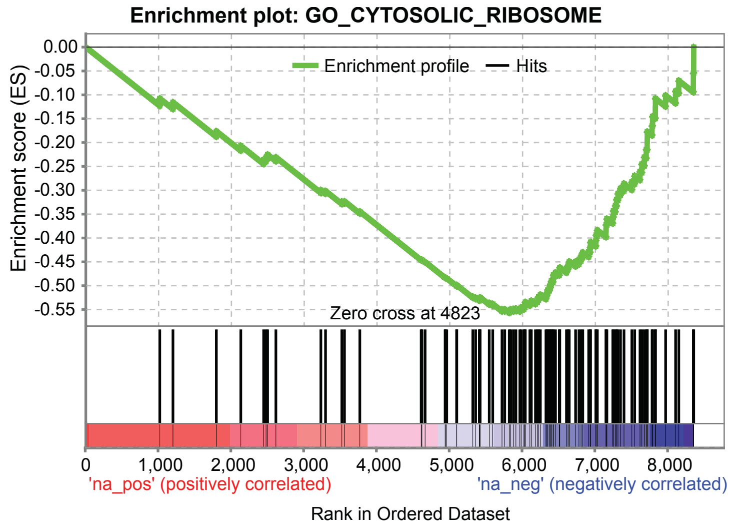

c)

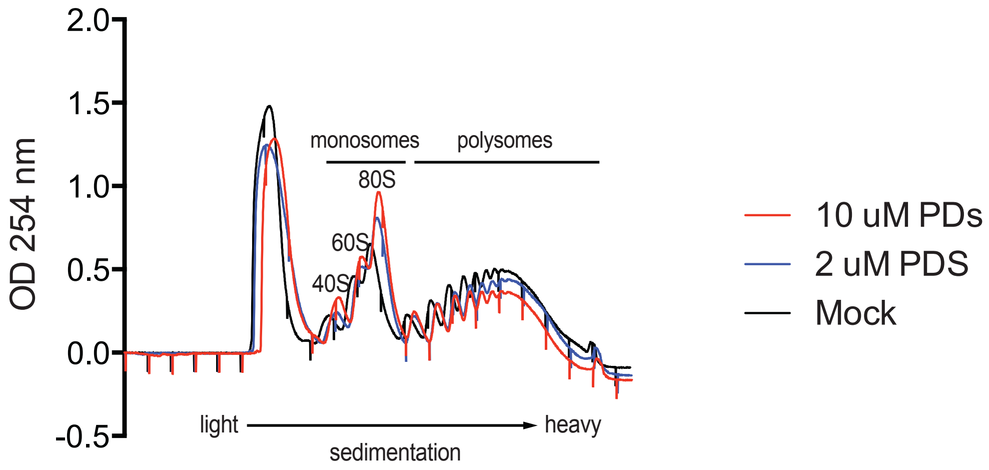

Figure. S12: PDS treatment reduces ribosomal protein levels measured by TMT proteomics. a) Western blotting for DNA damage marker γH2AX following treatment with DMSO or 2µM PDS. Treatment with cytotoxic agent etoposide (1µM) was used to elicit a DNA damage response. Treatment with translational inhibitor cycloheximide (10µg/ml) is included for comparison. Actin provides the loading control. b) Gene set enrichment analysis on dataset ranked for change in protein expression in cells treated with 2 µM PDS for 2 h compared to DMSO control quantified using tandem mass tag (TMT) based mass spectrometry. c) UV absorption at 254nM following sucrose fractionation for polysome profiling of cells treated with DMSO (black), 2µM PDS (blue) or 10µM PDS (red) for 45 mins. Monosomal and polysomal fractions are highlighted.

Table S1: RNA G4 motifs in ribosomal protein messenger RNA. Conservation of the predicted motif higher vertebrates is shown in bold.

Transcript	Species	RNA G4 Sequence
RPS19	Human	CUUUCCCCU**GG**CU**GG**CAGCGC**GG**A**GG**CCGCACG
	Chimpanzee	CUUUCCCCU**GG**CU**GG**CAGCGC**GG**A**GG**CCGCACG
	Pig	CUUUCCCCU**GG**CU**GG**CAGCGC**GG**A**GG**CCGCACG
	Dog	CUUUCCCCU**GG**CU**GG**CAGCGC**GG**A**GG**CCGCACG
	Mouse	CUUUCCCCU**GG**CU**GG**CAGCGC**GG**A**GG**CCGCACG
	Rat	CUUUCCCCU**GG**CU**GG**CAGCGC**GG**A**GG**CCGCACG
RPL12	Human	CTTTC**GG**CTC**GG**A**GG**A**GG**CCAA**GG**TGCAA
	Chimpanzee	CTTTC**GG**CTC**GG**A**GG**A**GG**CCAA**GG**TGCAA
	Pig	CTTTC**GG**CTC**GG**A**GG**A**GG**CTAC**GG**TGCAA
	Dog	CTTTA**GG**CTC**GG**A**GG**A**GG**CAAA**GG**TGCAA
	Mouse	CTTTC**GG**CTC**GG**A**GG**A**GG**CAAC**GG**TGCAA
	Rat	CTTTC**GG**TTC**GG**A**GG**A**GG**CAAC**GG**TGCAA
RPL13A	Human	CCGAAGAT**GG**C**GG**A**GG**TGCA**GG**
	Chimpanzee	CCGAAGAT**GG**C**GG**A**GG**TGCA**GG**
	Pig	CCGAAGAT**GG**C**GG**A**GG**GGCA**GG**
	Dog	CCGAAGAT**GG**C**GG**A**GG**GGCA**GG**
	Mouse	CCGAAGAT**GG**C**GG**A**GG**GGCA**GG**
	Rat	CCGAAGAT**GG**C**GG**A**GG**GGCA**GG**
RPS12	Human	CCGAGUCGCGC**GG**A**GG**C**GG**AGGCUU**GGG**
	Chimpanzee	CCGAGUCGCGC**GG**A**GG**C**GG**AGGCUU**GGG**
	Pig	CCGAGUCGCGC**GG**A**GG**C**GG**AGGCUC**GGG**
	Dog	CCGAGUCGCGC**GG**A**GG**C**GG**AAGCUCCU**GG**
	Mouse	CCGACUUGCGC**GG**A**GG**C**GG**AAGCUC**GGG**
	Rat	CCAAGUUCCGC**GG**A**GG**C**GG**AAGCUC**GGG**
RPL37A	Human	CUUUCU**GG**GCUC**GG**ACCUA**GG**UCGC**GG**CGAC
	Chimpanzee	CUUUCU**GG**GCUC**GG**ACCUA**GG**UCGC**GG**CGAC
	Pig	CUCUCU**GG**GCUC**GG**ACCG**GG**UUUGC**GG**CAAA
	Dog	CUCUCU**GG**GCUG**GG**ACCU**GG**CUGGC**GG**CGAC
	Mouse	CUCUUC**GG**ACUU**GG**GCUUG**GG**UUCC**GG**CGAC
	Rat	CUCUUC**GG**GCUU**GG**GCUUC**GG**UUCC**GG**CGAC
RPS11	Human	CUUUUUUUCA**GG**C**GG**CC**GGG**AAGAU**GG**C**GG**ACA
	Chimpanzee	CUUUUUUUCA**GG**C**GG**CC**GGG**AAGAU**GG**C**GG**ACA
	Pig	CCUUUUUUCUAGC**GG**CC**GGG**AAGAU**GG**C**GG**AUA
	Dog	CCUUUUUCCA**GG**C**GG**CC**GGG**AAGAU**GG**CAGACA
	Mouse	CCCUUUCUCC**GG**C**GG**CC**GGG**AAGAU**GG**C**GG**ACA
	Rat	CUUUUUCUCA**GG**CUGCC**GGG**AAGAU**GG**C**GG**ACA
RPL28	Human	**GG**CAAA**GG**TGTCGT**GG**T**GG**TCATTAAGC
	Chimpanzee	**GG**CAAA**GG**TGTCGT**GG**T**GG**TCATTAAGC
	Pig	**GG**CAAA**GGGG**TCGT**GG**T**GG**TGATGAAGC
	Dog	**GG**CAA**GGG**TGTGGT**GG**T**GG**TCCTGAAGC
	Mouse	**GG**CAAA**GGGG**TCGT**GG**TAGTTATGAA
	Rat	**GG**CAAA**GGGG**TCGT**GG**T**GG**TTATGAA
